# Analysis of surgical treatment of appendix neuroendocrine neoplasms—17 years of single-center experience

**DOI:** 10.1186/s12957-023-03025-6

**Published:** 2023-05-17

**Authors:** He-wei Zhang, Yi Jiang, Zhi-yang Huang, Xiao-cong Zhou

**Affiliations:** 1 Departments of Hepatobiliary and Pancreatic Surgery, The Dingli Clinical Institute of Wenzhou Medical University (Wenzhou Central Hospital), Wenzhou, Zhejiang Province People’s Republic of China; 2 Departments of Pathology, The Dingli Clinical Institute of Wenzhou Medical University (Wenzhou Central Hospital), Wenzhou, Zhejiang Province People’s Republic of China; 3 Departments of Gastroenterology, The Dingli Clinical Institute of Wenzhou Medical University (Wenzhou Central Hospital), Wenzhou, Zhejiang Province People’s Republic of China; 4 Departments of Colorectal Surgery, The Dingli Clinical Institute of Wenzhou Medical University (Wenzhou Central Hospital), Wenzhou, Zhejiang Province People’s Republic of China

**Keywords:** Appendiceal neoplasm, Neuroendocrine neoplasms, Pathology, Surgery

## Abstract

**Background/aim:**

This study investigated the clinicopathological characteristics and treatment of appendix neuroendocrine neoplasms in appendectomy specimens of our center.

**Materials and methods:**

The clinicopathological data, including age, sex, preoperative clinical manifestation, surgical method, and histopathological examination results of 11 patients with appendix neuroendocrine neoplasms confirmed by surgery and pathology between November 2005 and January 2023, were retrospectively analyzed.

**Results:**

In the histopathological examination of 7277 appendectomy specimens, 11 cases (0.2%) had appendix neuroendocrine neoplasms. Among the 11 patients, 8(72.7%) were males, and 3(27.3%) were females, with an average age of 48.1 years. All patients underwent emergency surgery. A total of 9 patients underwent open appendectomy, including 1 patient who underwent second-stage simple right hemicolectomy after an appendectomy, and two who underwent laparoscopic appendectomy. All 11 patients were followed up for a period of 1 to 17 years. All patients survived without any indication of tumor recurrence.

**Conclusion:**

Appendiceal neuroendocrine neoplasms are low-grade malignant tumors originating from neuroendocrine cells. They are rarely seen in clinical practice and are often treated based on acute and chronic appendicitis symptoms. These tumors are challenging to diagnose before surgery due to the lack of specificity in clinical manifestations and auxiliary examinations. The diagnosis generally depends on postoperative pathology and immunohistochemistry. Despite the diagnostic challenges, these tumors have a favorable prognosis.

## Introduction

Neuroendocrine neoplasms of the appendix, formerly known as carcinoids, are the most prevalent type of appendix tumors. They are rare tumors and grow slowly [1, 2]. The incidence rate of neuroendocrine neoplasms of the appendix is about 0.15–0.6/100,000/year. Western studies have revealed that the incidence of neuroendocrine neoplasms is higher in women than in men. The cancer is reported in individuals aged between 38 and 51 years old [3, 4]. The North American Neuroendocrine Society (NANETS) has established guidelines for classifying neuroendocrine neoplasms. Based on these guidelines, NENs are classified into highly differentiated and poorly differentiated categories. Carcinoid tumors, including low- and intermediate-grade tumors, are well-differentiated NENs. By definition, poorly differentiated NENs, including small-cell carcinomas and large-cell neuroendocrine carcinomas, are considered high-level neuroendocrine carcinomas. Cell size and nuclear morphology are used to distinguish small cell carcinoma from large cell neuroendocrine carcinoma. The mixed forms of advanced neuroendocrine and non-neuroendocrine carcinoma have also been well-recognized [5]. In contrast, the European Neuroendocrine Tumor Society (ENETS) grades tumors according to the histopathology characteristics of the appendix and Ki-67 proliferation index. Neuroendocrine neoplasms of the appendix are classified into three categories: NET-G1 (Ki67 index < 2%), NET-G2 (Ki67 index 3–20%), and NEC-G3 (Ki67 index > 20%) [3]. NEC-G3 includes small-cell carcinomas and large-cell neuroendocrine carcinomas. It can be seen that both ENETS and NANETS classify NEN into three hierarchical categories. However, the criteria for defining each category of NEN do not fully match between different guidelines.

In 2019, the World Health Organization classified the neuroendocrine tumors of the appendix into well‐differentiated neuroendocrine tumor (NET), poorly differentiated neuroendocrine carcinoma (NEC), and mixed neuroendocrine-non neuroendocrine neoplasms (MiNEN) [6]. NETs are relatively inert and have a good prognosis [7]. NETs can be characterized by cord-like, banded, and nested structures. Typical cells are circular to elliptical in shape, and the nucleus contains a rough collection of “salt and pepper” chromatin. Some tumors may show diffuse granular chromatin, and some may have prominent nucleoli. The cytoplasm usually has a strongly granular appearance [8, 9]. Neuroendocrine differentiation was observed using immunohistochemical synaptophysin (Syn) and chromaffin A (CgA) staining [3, 6]. NETs are further subdivided into G1, G2, and G3, according to the mitotic index and Ki-67 proliferative index. Among them, G1 and G2 have a Ki-67 proliferation index of < 20%, while G3 has a Ki-67 proliferation index of > 20%. G3 is relatively more invasive than G1 and G2. NEC is a highly invasive cancer (small or large cell carcinoma) with a poor prognosis (median survival rate of less than 2 years). Poorly differentiated neuroendocrine carcinomas show malignant cells arranged in sheets, with a high mitotic rate and conspicuous necrosis. Small cell neuroendocrine carcinoma shows intensely hyperchromatic cells that mold to one another, with minimal cytoplasm. Large cell neuroendocrine carcinoma shows cells with prominent nucleoli and some amount of amphophilic cytoplasm. NECs usually have TP53 or RB1 mutations [10]. MiNENs are tumors composed of a mixed population of neuroendocrine tumors and adenocarcinoma. Some studies have suggested that the two components account for at least 30% of the tumor component, but this viewpoint is still debatable [11].

Due to the lack of specific clinical symptoms for appendix neuroendocrine neoplasms, laboratory, B-ultrasound, X-ray, and other examinations are of little significance for the early diagnosis of these neoplasms. Preoperative clinical diagnosis is often misdiagnosed as other inflammatory lesions, most of which are found accidentally in the pathological examination of postoperative specimens [12–14]. So far, the biological behavior of neuroendocrine neoplasms has not been fully understood [15]. Some controversy still exists in the surgical treatment of neuroendocrine neoplasms of the appendix. Generally, tumor size is positively correlated with the chance of metastasis. For example, metastasis is rarely reported for tumors smaller than 1.0 cm. On the other hand, the chance of metastasis is high for patients with 1.0–2.0 cm tumors. However, whether radical surgery is needed for patients with 1.0–2.0 cm tumors remains controversial. Research by Moertel et al. showed that a simple appendectomy is an adequate treatment for patients with localized tumors smaller than 2.0 cm [16]. For tumors larger than 2.0 cm, the metastasis rate can reach 31%. Some studies have suggested that all patients with tumors larger than 2.0 cm should undergo radical surgery for the right colon [3, 17–19]. However, Moertel et al. suggested that the right hemicolectomy may only suit young patients with tumors ≥ 2.0 cm [16]. The neuroendocrine tumors of the appendix are rare, and the preoperative diagnosis is challenging. Therefore, it is crucial to retrospectively analyze the information about this rare tumor, which could reveal its appropriate treatment.

This study investigated the clinical data and histopathological characteristics of 11 cases of neuroendocrine neoplasms of appendix. The patients were treated at our center between November 2005 and January 2023. Moreover, we discussed the diagnosis, treatment, and prognosis of this cancer to offer reference and guidance for the clinical treatment of the cancer.

## Materials and methods

### Patients

A total of 7277 patients were retrospectively evaluated. All patients underwent appendectomy and histopathological examination at Wenzhou Central Hospital between November 2005 and January 2023. Only patients diagnosed with appendix neuroendocrine neoplasms were included in the study. The clinical data of patients were obtained from computer records and telephone follow-ups. The age, sex, preoperative clinical manifestation, surgical procedure, histopathology, and immunohistochemistry of these cases were recorded. If a patient met any of the following criteria, they underwent right hemicolectomy after simple appendectomy as part of their treatment: (1) the tumor diameter is greater than 2 cm; (2) tumor metastasis to the mesentery of the appendix; (3) mesenteric lymph node metastasis of the tumor; (4) tumor cells within the surgical margin of appendectomy; and (5) appendiceal NEC (grade 3, Ki67 > 20%). Patients were followed up once a year after surgery. The cancer recurrence was tracked for the remainder of their lives. The follow-up period was calculated as the time from diagnosis to recurrence or the last follow-up. The median follow-up time was 9.4 years (1–17 years). This study was approved by the ethics committee of Wenzhou Central Hospital.

### Hematoxylin and eosin staining and immunohistochemistry

The original specimen was examined and diagnosed by two experienced pathologists.

## Results

Among the 11 patients with neuroendocrine neoplasms of the appendix, 8(72.7%) were males, and 3(27.3%) were females, all with no family history of a malignant tumor. The patients ranged 9 to 71 years old, with an average age of 48.1 years. The main clinical manifestation in the 11 patients was acute appendicitis. The clinical and pathological data of the patients are shown in Table 1.

**Table 1:** Patient characteristics, treatment, pathological results, and immunohistochemical test results

	Category	***n***	Total (***n*** = 11) (%)
**Sex**	Male	8	72.7
	Female	3	27.3
**Age**	< 18	1	9.1
	≥ 18	10	90.9
**Auxiliary examination(B-ultrasound or CT)**	The appendix was slightly thickened	2	18.2
	Acute appendicitis with fecal calculus	2	18.2
	Acute appendicitis with fecal stone and peripheral exudation	1	9.1
	Acute appendicitis with peripheral exudation	3	27.3
	Acute appendicitis with perforation and peripheral exudation	1	9.1
	The possibility of inflammatory changes in appendicitis	1	9.1
**Surgical procedure**	Open appendicectomy	8	72.7
	Appendicectomy + right-sided hemicolectomy	1	9.1
	Laparoscopic appendectomy	2	18.2
**Size**	< 1 cm	9	81.8
	1–2 cm	2	18.2
	> 2 cm	0	0.0
**Extension**	Distal	8	72.7
	Body	3	27.3
	Proximal	0	0.0
**Tumor localization**	Mucosal layer	1	9.1
	Muscularis	6	54.5
	Serous layer	4	36.4
**Immunohistochemistry**	NSE(+)	9	100
	CgA(+)	9	100
	Syn(+)	9	100
**Ki 67%**	> 1%	0	0.0
	< 1%	9	100
**Mesoappendix invasion**	> 3 mm	0	0.0
	< 3 mm	11	100
**Vascular invasion**	Yes	0	0.0
	No	11	100
**Lymphatic invasion**	Yes	0	0.0
	No	11	100
**Perineural invasion**	Yes	0	0.0
	No	11	100
**Positive resection margin**	Yes	1	9.1
	No	10	90.9

### Supplementary examination

#### Imaging examination

Of the 11 patients with neuroendocrine tumors of the appendix, one did not undergo abdominal computed tomography (CT) or B-ultrasound examination before surgery. In contrast, the remaining 10 patients underwent abdominal CT or B-ultrasound examination before surgery (including 9 cases who underwent abdominal CT examination and 1 who underwent B-ultrasound examination). Among the 9 patients who underwent abdominal CT examination, 2 had a slightly thickened appendix, 2 had acute appendicitis with fecal calculus, one had acute appendicitis with fecal stone and peripheral exudation, 3 had acute appendicitis with peripheral exudation. One had acute appendicitis with perforation and peripheral exudation. B-ultrasound showed the possibility of inflammatory changes in appendicitis in one patient.

#### Laboratory examination

Preoperative blood routine examination of the 11 patients showed that the white blood cell count was normal or high (8.8~17.7 × 10^9^/L; normal range 3.5~9.5 × 10^9^/L). Because all patients were admitted to the hospital with acute abdominal pain, serum tumor markers were not examined before surgery.

### Pathological diagnosis and lymph node metastasis

**Fig. 1:**
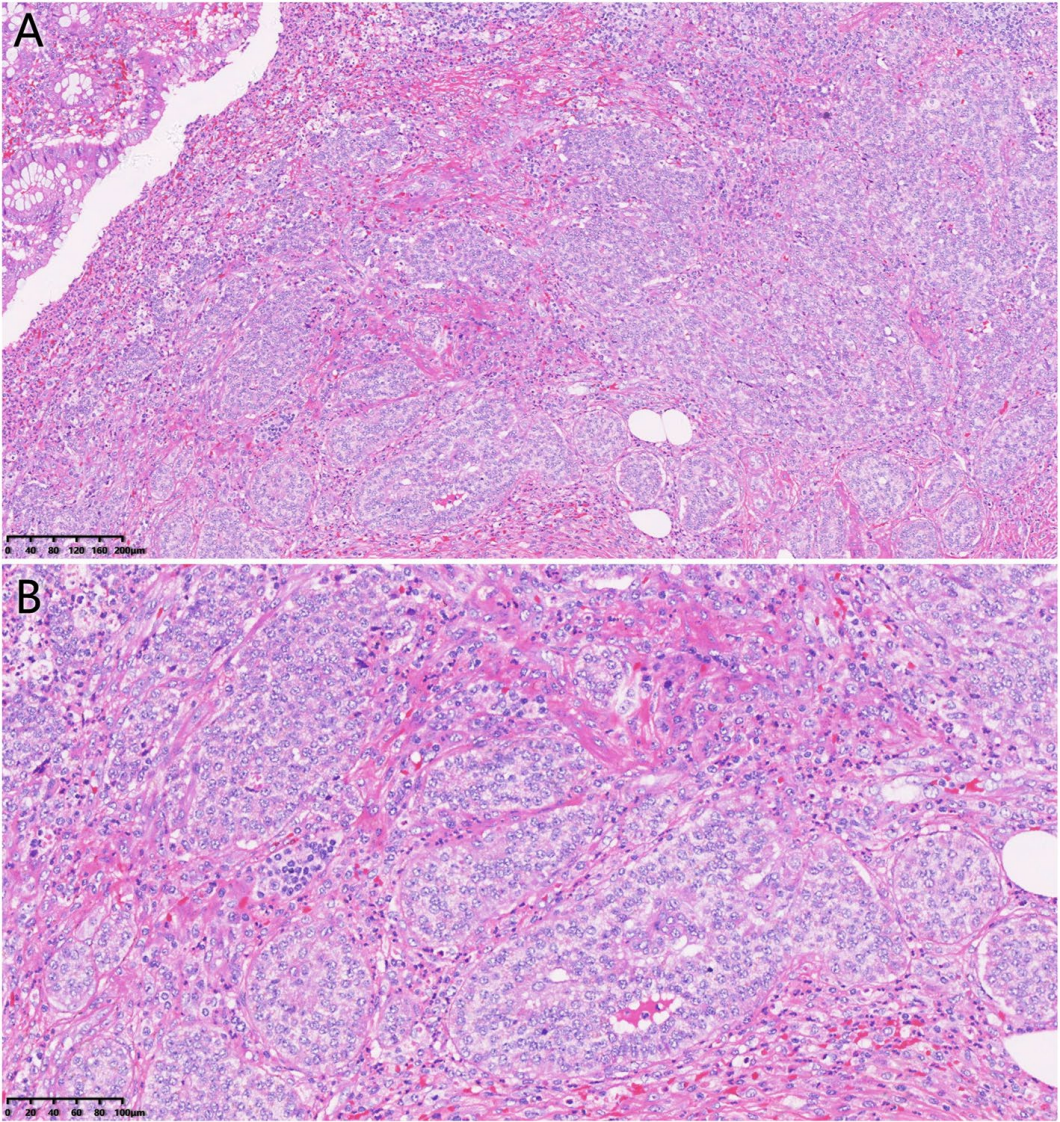
**A**, **B** Histopathological images of typical neuroendocrine neoplasm (G1) in a 66-year-old female patient. The lesion is located at the top of the appendix. The nucleus is round, uniform in size and staining, indistinct in boundary, and arranged in nests or bands. No vascular, lymphatic, or neural invasion was observed. There is no tumor invasion of the appendix mesentery. Tumor cells were not found at the surgical margin (hematoxylin and eosin stain; original magnification × 100 and × 200)

**Fig. 2:**
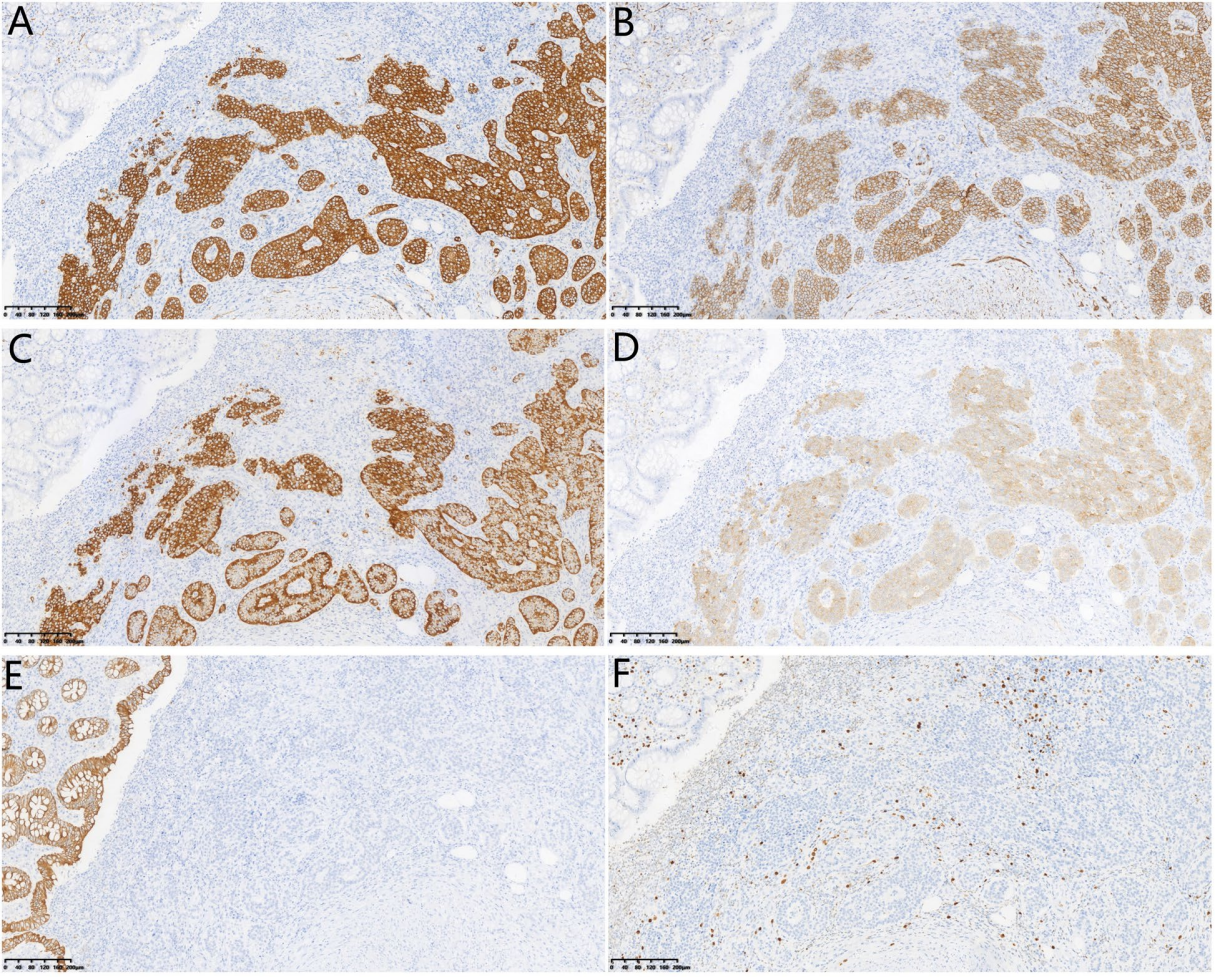
Immunohistochemistry staining of the neuroendocrine. **A** Positive staining for synaptophysin (Syn) (original magnification × 100). **B** Immunohistochemistry staining for neuroendocrine neoplasm positive for CD56 (original magnification × 100). **C** Immunohistochemical staining of neuroendocrine neoplasm positive for chromaffin A (CgA) (original magnification × 100). **D** Immunohistochemistry staining of neuroendocrine neoplasm positive staining for neuron-specific enolase (NSE) (original magnification × 100). **E** Immunohistochemical staining of neuroendocrine neoplasm negative for CK20 (original magnification × 100). **F** Immunohistochemical staining for Ki-67-positive cell population in neuroendocrine neoplasm. Ki-67-positive cells accounted for about 1% of the neuroendocrine neoplasm tissue (original magnification × 100)

Because all patients underwent emergency surgery, no intraoperative rapid frozen pathological examination was performed. About 72.7% of the tumors were located at the distal end of the appendix, while 27.3% were in the body. Of the tumors, 63.6% of tumors were less than 1 cm in diameter, and 36.4% were between 1 and 2 cm in diameter. The tumors were mainly located in the mucosal layer (9.1%), the muscular layer (54.5%), and the serous layer (36.4%). In one case, tumor cells were found at the surgical margin (9.1%). All patients had no vascular, lymphatic, or perineural invasion. Mesangial invasion of the appendix was absent in all patients. The histopathological images of typical neuroendocrine neoplasms cases are shown in Fig. 1. The nucleus is round, uniform in size and staining, indistinct in boundary, and arranged in nests or bands. Immunohistochemical staining of neuroendocrine markers such as neuron-specific enolase (NSE), chromaffin A (CgA), and synaptophysin (Syn), was performed for all cases, and all were positive (Fig. 2). The percentage of tumor ki67 activity was counted with the naked eye, which revealed that the average ki67 activity of all patients was less than 1%.

Postoperative pathological results showed that all the tumors in the 11 patients were neuroendocrine neoplasms of the appendix. Five patients had acute suppurative appendicitis, 3 had acute suppurative appendicitis with periappendicitis, and 2 had acute gangrenous appendicitis with perforation. One patient underwent a simple right hemicolectomy after appendectomy. Postoperative pathology revealed chronic inflammation of the right hemicolum with serosa chronic purulent inflammation, foam cell proliferation, and foreign body giant cell reaction. In addition, chronic inflammation was observed in 9 paraintestinal lymph nodes.

### Operation mode and postoperative complications

A total of 9 patients underwent open appendectomy. One of them had a neuroendocrine tumor of the appendix. At that time, peritonitis was caused by perforation of the appendix, and gangrene adhered the entire appendix segment to the surrounding tissues to form a mass. During the operation, the appendix was removed in sections, and it was impossible to determine whether tumor residue was present at the cutting edge. Therefore, a right hemicolectomy was performed. After an appendectomy, a pathological examination confirmed the presence of a neuroendocrine neoplasm of the appendix tip with a maximum diameter of 1.2 cm and which infiltrated the full thickness of the tube wall in one patient. However, the patient refused to undergo a secondary radical right hemicolectomy. Two patients underwent laparoscopic appendectomy. All patients recovered well without significant complications.

### Follow-up

Eleven patients were followed up for a median of 9.4 years (1–17 years). No recurrence or metastasis was observed during the follow-up period.

## Discussion

The neuroendocrine neoplasm of the appendix originates from the argyrophilic cells (also known as chromaffin cells) in the lower skin of the appendix mucosa, also known as an argyrophilic tumor. Its incidence rate is low, with a high clinical misdiagnosis rate due to a lack of specific clinical manifestations [20]. The diagnosis depends on the intraoperative frozen section, postoperative pathological paraffin and immunohistochemical examination. The neuroendocrine neoplasms of the appendix account for 50~70% of all appendix tumors [21]. Symptoms occur in patients with an average age of 49 (age distribution is 0–85 years old) [22]. The neuroendocrine neoplasms of the appendix are more prevalent in women than in men [15, 23]. To date, the etiology of neuroendocrine carcinoma of the appendix remains unclear. Recent reports have indicated that endometriosis may be related to the occurrence of neuroendocrine carcinoma of the appendix [24, 25]. Virgine C et al. described five children with neuroendocrine tumors of the appendix (NET) associated with parasitic intestinal infections and discussed the possibility of inflammation leading to canceration. However, the number of cases was still small [26]. The onset age of the patients in our cohort was 9 to 71 years old, with an average age of 48.1 years, consistent with previous studies. However, the ratio of women to men in this group was 3:8, inconsistent with previous studies.

Diagnosing neuroendocrine tumors of the appendix before surgery is challenging due to the lack of specificity in clinical manifestations and auxiliary examinations. As a result, the cancer is detected in most patients unexpectedly during appendectomy [27]. In our cohort, all patients except one who received treatment for acute appendicitis were diagnosed with neuroendocrine neoplasms during their appendectomy for acute appendicitis. Coursey et al. demonstrated the challenge of detecting appendix tumors using CT scans [28]. Kangaspunta et al. emphasized that CT cannot exclude the tumor etiology of acute appendicitis [29]. Among the 9 patients who underwent abdominal CT examination in our cohort, 2 showed only slight thickening of the appendix, 2 showed acute appendicitis with fecal stone, 1 showed acute appendicitis with fecal stone and peripheral exudation, 3 showed acute appendicitis with peripheral exudation, and 1 showed acute appendicitis with perforation and peripheral exudation. B-ultrasound examination suggested appendicitis in one case. The preoperative imaging examination of these 10 patients failed to indicate the possibility of a malignant tumor of the appendix (one patient did not undergo abdominal CT or B-ultrasound examination before surgery). In the laboratory examination, the preoperative blood routine examination of our cohort showed that the white blood cell count was normal or high (8.8~17.7 × 109/L, normal range 3.5~9.5 × 109/L), which is the same as the blood routine of general acute and chronic appendicitis. Since all patients were admitted to the hospital with abdomen pain needing emergency surgery, serum tumor markers were not examined before surgery.

Diagnosing neuroendocrine tumors of the appendix mainly depends on postoperative pathological paraffin sections and immunohistochemistry. Highly differentiated neuroendocrine tumors (NET) of the appendix have small and consistent round or small polygonal tumor cells arranged into solid nests or islets under the microscope, sometimes with tubular chrysanthemum structures. Mitotic figures are very rare. Immunohistochemistry of tumor cells has a different expression of neuroendocrine markers, including CgA, Syn, and NSE [30, 31]. NEC and MiNENs are invasive tumors usually diagnosed in advanced stages and have poor prognoses.

On the other hand, NETs are relatively inert and have a good prognosis [7]. NETs are further subdivided into G1, G2, and G3, according to the mitotic index and Ki-67 proliferative index. Of the 11 cases of neuroendocrine neoplasms of the appendix, all patients, except for 2 cases without immunohistochemical examination, were CgA and Syn positive (including 7 cases with NSE positive), consistent with the diagnostic criteria of neuroendocrine tumors. Of the 11 cases of neuroendocrine neoplasms of the appendix, all patients, except for 2 cases without immunohistochemical examination, were CgA and Syn positive (including 7 cases with NSE positive), consistent with the diagnostic criteria of neuroendocrine tumors.

The neuroendocrine neoplasms of the appendix are generally small, mainly located in the submucosa, most of which are less than 2 cm in diameter. Furthermore, neuroendocrine neoplasms of the appendix were only detected under the microscope [32]. Surgical resection is the main method for appendix neuroendocrine neoplasms, and the surgical method is selected according to the size, location, invasion, and metastasis of the lesion. For tumors with the diameters of neuroendocrine neoplasms at the tip and middle segment of the appendix less than 1 cm, the tumors are mostly confined to the serous membrane, with almost no distant metastases. Simple appendectomy can improve the prognosis [16, 33, 34]. However, for neuroendocrine neoplasms of the appendix root with a diameter of less than 1 cm, especially for young patients, ileocelectomy should be selected [35]. The distant metastasis rate is high for appendix neuroendocrine neoplasm with a diameter > 2 cm. Generally, radical right hemicolectomy should be performed [36, 37]. There is still controversy about the surgical method for the neuroendocrine neoplasms of the appendix with a diameter of 1–2 cm. ENETS guidelines recommend right hemicolectomy for tumors 1 to 2 cm wide with appendiceal media infiltration, positive margins, vascular infiltration, and Ki-67 labeling index > 2%. Some studies have suggested the removal of the appendix and complete removal of the mesenteric portion of the appendix [35]. The guidelines of the NANETS recommend that patients with tumors larger than 2 cm, patients with incomplete resection of the tumor, or patients with appendiceal carcinoid tumors that spread to lymphatic vessels, appendix media, and moderate to severe tumors undergo right hemicolectomy [5]. As for neuroendocrine carcinoma (NEC) with a high degree of malignancy, Holmager, P. et al. suggested that surgery should be expanded beyond the local disease and a curative surgery should be performed [38]. Advanced mixed neuroendocrine nonneuroendocrine tumors (MiNEN) have neuroendocrine and non-neuroendocrine components. Most MiNENs have poor prognoses, and the benefits of surgery are unclear.

Pawa et al. pointed out that more than 90% of the appendix NETs are located at the distal end of the appendix. According to a review published in 2018, NETs located at the distal end of the appendix account for 60–75% of the most common location of the appendix [39]. Herein, 8 NETs were located at the appendix tip, 3 at the body, and none at the root of the appendix. Of the 11 study participants, 7 had tumor lesions < 1 cm, 4 were within the 1–2 cm range, and none had a diameter > 2 cm. One patient underwent second-stage simple right hemicolectomy after appendectomy due to peritonitis caused by appendix perforation, gangrene of the whole segment, and adhesion to surrounding tissues. Because the appendix was removed in segments during the operation, determining tumor residue at the cutting edge was challenging. The other 10 cases underwent simple appendectomy (including 2 cases of laparoscopics appendectomy). Immunohistochemistry showed that Ki67 was less than 1%. Considering the low risk of malignancy, the second stage right hemicolectomy was not performed after the appendectomy, and close outpatient follow-up was required after surgery. One case of neuroendocrine neoplasm at the tip of the appendix with the largest diameter of 1.2 cm and infiltrating into the full thickness of the tube wall was recommended to undergo secondary radical right hemicolectomy. However, because the patient refused to be reoperated, he was advised to attend regular outpatient follow-up check-ups, including colonoscopy and abdominal CT scans. In the present study, 11 patients with neuroendocrine tumors of the appendix were followed up for a long time after surgery, and all of them survived without tumor recurrence or metastasis, indicating that the prognosis of the neuroendocrine tumor of the appendix was good after surgical treatment.

This study had several limitations. First, our study was a single-center retrospective analysis. In addition, although this study included all cases of appendiceal neuroendocrine tumors in our center, the sample size was still small due to the low incidence of this disease. Therefore, further large-scale multicenter prospective studies of appendix neuroendocrine tumors are needed.

## Conclusion

In summary, the incidence rate of neuroendocrine tumors is low and the tumors are usually treated as acute and chronic appendicitis symptoms. In addition, it is challenging to make an early diagnosis due to the lack of specific clinical manifestations and auxiliary examinations. Regarding treatment options, we recommend simple appendectomy for localized neuroendocrine tumors of the appendix smaller than 2 cm. However, we recommend additional right hemicolectomy for a favorable patient outcome for tumors larger than 2 cm or tumors accompanied by lymphatic, vascular, perineural, and appendiceal media invasion.

## Data Availability

All data generated or analyzed during this study are included in this published article. Further information can be obtained from the corresponding author on reasonable request.
